# Supplementary material for: A UMLS-based spell checker for natural language processing in vaccine safety
Source: BMC Med Inform Decis Mak. 2007 Feb 12;7:3. doi: 10.1186/1472-6947-7-3 (PMC1805499; doi:10.1186/1472-6947-7-3)
Supplement: Additional file 13 — RAP application directory. Sets up directory for RAP (RDF application for PHP) source code [file 1472-6947-7-3-S13.gz › rap/doc/tutorial/rdql_tutorial.htm]

RDQL Tutorial


### RDF API for PHP V0.9.1

# RDQL Tutorial

This turorial is part of the RAP - Rdf API for PHP documentation.

  

Radoslaw Oldakowski <radol@gmx.de>   
October 2004

  

In this tutorial I will explain the syntax of the RDQL query language implemented in RDF API for
PHP and show how to query RDF models created within the API. The exact RDQL grammar
for RAP's RDQL Engine, described in Bacus Naur Form (BNF), is provided here.

The general form of an RDQL query, shown below, resembles an SQL statement:

`SELECT variables listing   
FROM rdf documents   
WHERE patterns   
AND filter expressions   
USING prefix declaration`

  
  

### SELECT

Every successful query returns a set of variable bindings. In the `SELECT` clause we define a list of variables to be returned.
Each variable is introduced by a question mark (?) and can be named using alphanumerical characters combined with an underscore (\_).
Multiple variables are separated by a space and/or an optional comma (,), for example:

`SELECT ?name ?email, ?age,?tel_number`

In case that we want to specify all query variables, we can use the SQL-like shortcut in form of a star (\*):

`SELECT *`

  
  

### FROM

In the `FROM` clause we specify the path or URL of the RDF document
to be queried. However, this tutorial focuses on querying RDF models created
within RAP (MemModels or DbModels) by passing the RDQL query string to the method
`rdqlQuery()`. In this case the query is performed on a particular
model, thus there is no need to specify it once again. If the passed string
contains a `FROM` clause, it will then be ignored by the query engine.

  

### WHERE

In the `WHERE` clause we indicate a list of triple patterns which
have to be matched by each valid query result set. All patterns representing
an RDF statement have the form `(subject, predicate, object)` where
the subject, predicate, and object can either be a `<URIref>`
or a `?variable`. The object can moreover be a `"Literal"`,
for example:

`WHERE (?resource, <http://www.w3.org/2001/vcard-rdf/3.0/EMAIL>, "radol@gmx.de")`

This will match any statement of an RDF model with a predicate indicated
by the above URIref (vCard's email property) and an object represented by
the literal `"radol@gmx.de"`. Furthermore, we can specify the language
and datatype of a literal:
`"literal string"@lang^^<datatypeURI>`. Note that the language
tag has no effect when the literal is datatyped, except for `rdf:XMLLiterals` and
plain literals. Correspondingly to the `SELECT` clause, using commas
(,) is also optional.

  

### AND

In addition to the triple patterns of the `WHERE` clause, in the
`AND` part of an query we can specify Boolean expressions over values
of URIs and literals. RAP's implementation of RDQL shows the real expressive
power of this query language. And so, we can use:

- arithmetic conditions (including multiplicative and additive operators), for instance:

`AND (10 + ?age)*2 <= 60/2, ?weight == 75`

(multiple expressions are separated by commas)

- string equality expressions, where `EQ` stands for "equal" and `NE` for "not equal":

`AND ?name EQ "Radoslaw Oldakowski", ?resource NE <http://example.org/>`

  
- Perl-style regular expressions, where "~~" | "=~" stands for "match"
  and "!~" for "not match", for example, return only HTML documents:

`AND ?document ~~ "/\.html$/i"`

We can also combine multiple filter expressions using logical operators "&&" (AND), "||" (OR):

`AND ( (10 + ?age)*2 <= 60/2) && (?name EQ "Radoslaw Oldakowski") )`

as well as use negation indicated by the exclamation mark (!):

`AND !( ((10 + ?age)*2 <= 60/2) && (?name1 EQ ?name2) )`

  
  

### USING

To make the query easier to read and write for humans, RDQL provides a way
to shorten the length of URIs (used in the `FROM, WHERE`
and `AND` clauses) by defining a string prefix. Every prefix is
defined in the `USING` clause as demonstrated below:

`WHERE (?resource, vCard:EMAIL, "radol@gmx.de")   
USING vCard FOR <http://www.w3.org/2001/vcard-rdf/3.0/>`

Note that since RAP V0.8 QNames (e.g. vCard:EMAIL) in contrast to full URI's
need not to be surrounded by '<' and '>'. However, the RDQL parser is also
backawrd compatible
with earlier versions of the RDQL grammar (RAP V0.6 - RAP V0.7.1). Moreover,
RAP supports default prefixes (e.g. `rdf`, `rdfs`, `xsd`)
that can be used without an explicit definition.

  

### Comments

While writing very long and complicated RDQL queries, it is often of great benefit to comment the code. For this purpose RAP allows us to use two kinds
of comment syntaxes. The first one is the one-line comment introduced by "//", the second has the form of `/* this is a comment */`.

  

### Query Examples

At this point it is time to put together all the pieces explained so far and
present RAP's RDQL Engine in action. In the following part of this section we
will be querying an RDF document describing employees of an example corporation
(employees.rdf). The working code for the examples
that will be demonstrated in this RDQL tutorial is rdql\_tutorial.php.

We shall start with including all RAP classes needed and loading the document into a MemModel:

`// Include all RAP classes   
define("RDFAPI_INCLUDE_DIR", "C:/Apache/htdocs/rdf_api/api/");   
include(RDFAPI_INCLUDE_DIR . "RdfAPI.php");   
  
// Create a new MemModel and load the document  
$employees = ModelFactory::getDefaultModel();  
$employees->load(RDFAPI_INCLUDE_DIR ."employees.rdf");`

Once the model has been created, we can perform our first query which will be: "*find the full name of all employees*".
So, we create a variable `$query1` and assign it the corresponding query string:

`$query1 = '   
SELECT ?fullName   
WHERE (?x, vcard:FN, ?fullName)   
USING vcard FOR <http://www.w3.org/2001/vcard-rdf/3.0#>';`

To execute the query, we simply call the method `rdqlQuery()` on the MemModel and pass the variable holding the query string as parameter:

`$result1 = $employees->rdqlQuery($query1);`

The result returned is an array of variable bindings with the values of these variables being RAP objects (`Resource`,
`Literal` or `BlankNode`). This enables us to use these objects for model update or other API calls.
We can, of course, print out the result on the screen as well. For this purpose, RAP's RDQL engine offers a very convenient method
`writeQueryResultAsHtmlTable()`. All we have to do is to pass the query result to this method:

`RdqlEngine::writeQueryResultAsHtmlTable($result1);`

This will result in the following output in the browser window:

|  |  |
| --- | --- |
| **No.** | **?fullName** |
| 1. | Literal: Bill Parker |
| 2. | Literal: George Simpson |
| 3. | Literal: Monica Murphy |

If we did not want to have objects being returned as variable values but their string serialization instead,
we would call the method `rdqlQeury()` and pass `FALSE` as the second parameter:

`$result2 = $employees->rdqlQuery($query1, FALSE);`

Note that in this case the resulting array could not be passed to the method `writeQueryResultAsHtmlTable()` so that we had to iterate it
using PHP's built-in `foreach` construct.

As you can see querying RDF models in RAP is extremely easy. Similarly we also query persisted models by passing the RDQL string to the method
`rdqlQuery()` called on a DbModel. Hence, from this point on I will only explain how to formulate a query and show its corresponding result.

In our next query: "*find the given name of all employees over 30*" we have to use a filter expression.
Moreover, in order to find out the given name we must specify the corresponding path in the graph leading through a Blank Node (marked bold):

`SELECT ?givenName, ?age   
// This is an example of an one-line comment   
WHERE (?x, vcard:N, ?blank),   
      (?blank, vcard:Given, ?givenName),   
      (?x, /* and this is another type
of comments */ v:age, ?age)   
AND ?age > 30   
USING vcard FOR <http://www.w3.org/2001/vcard-rdf/3.0#>
v FOR <http://sampleVocabulary.org/1.3/People#>`

In the above example, I have also demonstrated a possible way to insert comments. Here, the variable `?blank` is only used to link the resource
to the given name. We did not specify `?blank` in the `SELECT` clause, consequently we get:

|  |  |  |
| --- | --- | --- |
| **No.** | **?givenName** | **?age** |
| 1. | Literal: Bill  Literal: 33 **(rdf:datatype="http://www.w3.org/TR/xmlschema-2/integer")** | |  |
| 2. | Literal: George  Literal: 41 **(rdf:datatype="http://www.w3.org/TR/xmlschema-2/integer")** | |  |

Note that the value of `?age` is a datatyped literal. Yet another even more complex example might be: "*find the private telephone number
of the person whose office number is '+1 111 2222 668' and additionally return his given name and age*". In this query we have to specify several paths in the graph.
Furthermore, we use a string equality expression in the `AND` clause to filter the office number:

`SELECT ?givenName ?age ?telNumberHome  
WHERE (?person vcard:N ?blank1)  
      (?blank1 vcard:Given ?givenName)  
      (?person v:age ?age)  
      (?person vcard:TEL ?blank2)  
      (?blank2 rdf:value ?telNumberHome)  
      (?blank2 rdf:type vcard:home)  
      (?person vcard:TEL ?blank3)  
      (?blank3 rdf:value ?telNumberOffice)  
      (?blank3 rdf:type vcard:work)  
AND ?telNumberOffice eq "+1 111 2222 668"  
USING vcard FOR <http://www.w3.org/2001/vcard-rdf/3.0#>  
      v FOR <http://sampleVocabulary.org/1.3/People#>`

As shown in this example we can leave out commas. Note also that we make use of the default prefix `rdf`
(e.g. `rdf:type`) supported by RAP's RDQL engine. This query returns one matching set of variable bindings:

|  |  |  |  |
| --- | --- | --- | --- |
| **No.** | **?givenName** | **?age** | **?telNumberHome** |
| 1. | Literal: Bill  Literal: 33 **(rdf:datatype="http://www.w3.org/TR/xmlschema-2/integer")**  Literal: +1 111 2212 431 | | |  |  |

So far, the values of all variables returned were literals. However, we can ask for resources as well. Consider the following query:
"*find the resources that represent employees whose family name starts with 'M' and additionally return the corresponding office email address*".
This can be written in RDQL as shown below:

`SELECT ?resource, ?email   
WHERE (?resource, vcard:N, ?blank1)   
      (?blank1, vcard:Family, ?familyName)   
      (?resource, vcard:EMAIL, ?blank2)   
      (?blank2, rdf:value, ?email)   
      (?blank2, rdf:type, vcard:work)   
AND ?familyName ~~ "/^M/"   
USING vcard FOR <http://www.w3.org/2001/vcard-rdf/3.0#>`

In this case we use a regular expression to indicate the first letter of the family name
and once again apply the default prefix `rdf`. This results in:

|  |  |  |
| --- | --- | --- |
| **No.** | **?resource** | **?email** |
| 1. | Resource: http://example.com/employees/MonicaMurphy/  Literal: M.Murphy@example.com | |  |

For more query examples see Documentation
included in RAP documentation. You can additionally visit the website with the
Interactive
RDQL Demonstration allowing you to practice with your own documents and
queries. Furthermore, 
RDQL Test Cases give you an even deeper insight into RAP implementation
of RDQL by providing the opportunity to go through 77 queries testing different
aspects of the language implementation, performed in real time on both a memory
and a persisted model.
